# Book Reviews

**DOI:** 10.1038/sj.bjc.6690259

**Published:** 1999-03


*British Journal of Cancer* (1999) **79**(9/10), 1629–1631
© 1999 Cancer Research Campaign
Article no. bjoc.1998.0259

### Book Reviews

### Surgery for Bone and Soft Tissue Tumours

MA Simon and D Springfield.
pp 756, ISBN 0-397-51396-8.
Lippincott-Raven Publishers, USA

Orthopaedic oncology is a relatively new specialty which has evolved rapidly in the past 25 years thanks to advances in diagnostic imaging, chemotherapy, surgical techniques and reconstructive methods. The pioneer in establishing orthopaedic oncology as an international specialty was, without doubt, William Enneking, who continues to be active teaching, guiding and instructing throughout the world. This book has been written by his disciples and is a distillation of the last 25 years' experience with a strong emphasis on the North American experience. The two Editors both have impeccable pedigrees and have written widely on the subject, as have their seven assistant Editors and most of the 54 contributors, of whom only three do not come from North America.

Despite its North American bias, this book is well written and beautifully illustrated. It takes the reader through the journey from presentation, through diagnosis, to a whole range of surgical treatments for both benign and malignant conditions. The book is well-structured with the first section discussing the basic science and general principles of treatment; there are then further sections dealing with benign bone tumours, malignant bone tumours, soft tissue tumours and, finally, metastatic disease presenting in bones. All of the chapters are written by recognized authorities.

As a recipe book this is one of the few texts currently available to offer a broad review of the various options for surgical management of any one condition, both in terms of diagnosis and in terms of location. There are clear line drawings detailing how to do some of the more complicated operations and sensible advice about management.

It is clearly recognized from every chapter that the first role of the Orthopaedic Oncologist is to adequately resect the tumour, and considerable emphasis is placed on this. The second part of the job is the reconstructive process, and this is well addressed in most chapters with an emphasis often being determined by the individual author's preference. As such, one of the deficiencies is the absence of a well-argued case posing the benefits and disadvantages of various types of reconstruction. To a certain extent this is not surprising as even long-term comparisons have, in some instances, failed to show clear cut differences between prosthetic replacements, allografts and other forms of reconstruction.

This book does not take the place of adequate training in orthopaedic oncology and, as such, should not be used by the occasional operator. It is a book that is an essential complement to any Orthopaedic Oncology Department and will also be useful for anyone dealing with soft tissue sarcomas or with a special interest in metastatic disease to bone. Given the rarity of bone and soft tissue sarcomas, and the increasing recognition that centralization of treatment is important, this book is unlikely to have a large circulation, but those who use it will value it greatly.

One omission I would like to see corrected in future volumes is the lack of any detailed discussion about amputations and outcomes following this form of treatment. The elegant and complex operations described in this book may appear attractive to both surgeon and patient, yet a straightforward amputation may be oncologically and functionally better in many situations and this alternative should never be forgotten.

*RJ Grimer*
*Royal Orthopaedic Hospital Oncology Service,*
*The Royal Orthopaedic NHS Trust,*
*Woodlands, Northfield,*
*Birmingham*
*B31 2AP, UK*

### Detection and Treatment of Breast Cancer

I Fentiman.
Publication details: pp. 353
Price £59.95, ISBN 1-85317 2235.
Publisher Martin Dunitz Publishers

It was a pleasure to review this book for two main reasons. First, unlike the vast majority of modern texts it is written by a single author and therefore brings a very personal view of his expertise to the reader. Secondly, one reads the book in the knowledge that the author is an active specialist in breast disease and that the opinions given are based on true experience in clinical practice.

There are 24 chapters covering all aspects of breast cancer, with the exception of metastatic disease. It is therefore very much a book for clinical surgeons. Each of the chapters is clearly written and is extremely well supported by an exhaustive list of references. The references, I am pleased to note, list data from many international centres and do not concentrate solely on the North American literature, which is a great limitation of a number of American texts.

Inevitably, there are the occasional weaknesses. For example, the chapter on breast reconstruction might be regarded as being somewhat brief for 1998. Similarly, psychological aspects of breast cancer are rather scantily addressed.

Despite the above minor criticisms, and the fact that this very individual book is occasionally somewhat iconoclastic, I found it a most informative and enjoyable text to read and would recommend it to both libraries and any surgeon with an interest in breast disease.

*M Greenall,*
*Department of Surgery,*
*Oxford Radcliffe Hospital,*
*Level 2, Headley Way,*
*Headington,*
*Oxford OX3 9DU, UK*

### Advances in Head and Neck Oncology

Edited by K Thomas Robbins.
Publication details: pp. 181, 36 ill, £67.50,
ISBN 1-56593-840-2. Singular Publishing Group:
San Diego, CA

This little book aims to assist head and neck cancer 'care providers' to keep up-to-date on new developments in the field. It highlights 12 areas in which significant advances have been made over the past few years. Five of these concern surgical techniques.

There is a chapter entitled 'Biology of upper aerodigestive tract carcinoma' that succinctly but comprehensively covers the topics of tumour cell kinetics, apoptosis, regulation of proliferation and mechanism of invasion, with over 100 references. The author concludes that 'molecular advances in the field of gene therapy show great potential for improving disease control'. The reader is left reflecting that this potential is yet to be realized.

There is a brief but comprehensive review of hyperfractionated and accelerated radiotherapy for head and neck squamous carcinoma, similar to many others that have appeared in the literature over the past few years. The section on rhabdomyosarcoma of the head and neck in children is a good, balanced review of the subject. The author rightly warns against the use, outside of a research setting, of the chemotherapy-only approach now in vogue, stating that the desire to avoid radiotherapy risks a higher local recurrence rate and, consequently, the need for more mutilating salvage surgery or high-dose chemotherapy.

The editor describes his latest results of high-dose intra-arterial cisplatin concomitant with radiotherapy for advanced disease. He claims a loco-regional control at 30 months of 85% in 78 patients, without so far observing enhanced morbidity, making out a good case for further investigation of this approach.

A concise account of functional assessment of patients treated for head and neck cancer describes simple and straightforward methods for assessing the impact of treatment on patients' way of life. Perhaps significantly, this follows two chapters about surgical management of early laryngeal cancer, in which the authors appear completely to discount radiotherapy as an alternative approach. By way of contrast, the last chapter in the book, entitled 'Quality of life, co-morbidity and cost-effectiveness in head and neck cancer treatment', describes in detail the various methods that have been used to 'measure' quality of life in head and neck cancer patients. One is left wondering how much reliance can be placed on studies that regard symptoms scores as measurements to which parametric statistical tests can be applied.

The price is modest, so this book should find a place in the libraries of head and neck oncology centres.

*Dr JM Henk*
*Dept of Radiotherapy & Oncology*
*The Royal Marsden Hospital*
*Fulham Road*
*London SW3 6JJ*

### Prognostic and Predictive Value of p53

Edited by JGM Klijn.
Publication details: pp. 174, price hardbound $95,
NGL 160.00 ISBN 0444 82832X
European School of Oncology Scientific Updates, 1

This volume heralds the first in a new series from the European School of Oncology which tackles timely issues in oncology and aims to produce a rapid, state of the art book which will be of interest to both pre-clinical and clinical researchers, as well as to students. Not surprisingly, p53 was selected as the first subject in view of its important role in the development and progression of cancer and its implications for treatment.

The book was conceived as the final aim of a Task Force Meeting that took place in London on 3–4 December 1996. This meeting discussed a variety of aspects of p53, but focused on the predictive value of p53 regarding the response to different treatment modalities and its role in treatment resistance. The papers were written in 1997.

The book consists of ten chapters, six of which are concerned with breast cancer. The topics include the significance of different subgroups of p53 mutation, the prognostic and predictive significance of p53 determined by mutation analysis, immunohistochemistry and luminometric immunoassay, the impact on response to therapy, its relationship and interaction with other factors, its role in angiogenesis, its role in breast cancer risk assessment and its potential as a target for gene therapy. The first chapter sets the style of the book; it is a reader-friendly background to p53, written by Thierry Soussi, which gives a taste of some of the major areas where p53 may have clinical impact. The remaining chapters are interspersed with a significant amount of new or unpublished data alongside extensive reviews of the literature. Indeed, considerable effort has been spent in collating the wealth of information to be found in the scientific literature. This has resulted in several informative tables that will be of help and interest to researchers in the field, as well as to clinicians who wish to understand the potential value of p53.

The book highlights the contention that surrounds the clinical utility of p53. The lack of consensus in the scientific literature is mirrored in the book. A chapter by Anne-Lise Borreson-Dale describes the role of p53 as pivotal in determining biological behaviour of breast cancer and that it may accurately predict the response to different therapeutic interventions. Richard Elledge is more cautious and suggests it is unclear whether p53 will provide additional information to predict response to systemic therapy in breast cancer. The consequence is that there is no clear message as to the definitive role for p53 in the clinical management of the cancer patient. However, this is the correct interpretation of the

*© Cancer Research Campaign 1999*

current state of knowledge. Much more research will be required, including well-designed prospective clinical trials and accurate and reproducible methods to assess p53 function, before clinical decisions can be made based on the status of this gene alone or in combination with other factors. It is clear that p53 has not yet made the transition from a prognostic to a predictive marker but the promise for future developments remains encouraging.

The book will be a useful addition to people, both in and on the periphery, of p53 research in oncology. As with all books of this type, there is scope for improvement. There is certainly a degree of repetition between the chapters in describing the basic concepts and biological properties of p53. It would have been germane for the editor to finish with a final summary where the relevant information was extracted from each chapter and incorporated into a global and informed view. Even this book will be rapidly out of date due to constant accumulation of new knowledge in the p53 field. However, this series looks set to provide interesting future publications and it would be propitious to revisit this particular subject in the not too distant future.

*GD Wilson,*
*CRC Gray Laboratory*
*PO Box 100*
*Mount Vernon Hospital*
*Northwood*
*Middlesex HA6 2JR*

*© Cancer Research Campaign 1999*

